# India Multi- Regional Input-output Dataset for 32 States from 2011 to 2019

**DOI:** 10.1038/s41597-026-07227-x

**Published:** 2026-04-24

**Authors:** Heran Zheng, Jie Li, Yazhuo Lu, Wenqiang Wang, Zhenyu Wang, Jing Meng, Dabo Guan

**Affiliations:** 1 The Bartlett School of Sustainable Construction, University College London, London, WC1H 0QB UK; 2 Institute of Blue and Green Development, Shandong University, Weihai, China; 3 Department of Civil Engineering, The University of Hong Kong, Pok Fu Lam, 999077 Hong Kong; 4 Department of Earth System Science, Ministry of Education Key Laboratory for Earth System Modelling, Tsinghua University, 100084 Beijing, China

**Keywords:** Geography, Developing world

## Abstract

India has become increasingly integrated into global production networks over the past decade, accompanied by rapid transformation of its domestic economic system. The multi-regional input–output (MRIO) model is a widely used tool for describing intersectoral supply chains within complex economic systems. However, a comprehensive MRIO model for one of the world’s largest emerging economies has been lacking. Here, we develop a time-series MRIO dataset for 32 Indian states covering 2011–2019, comprising 66 sectors and five final demand categories. Using a hybrid approach that integrates multiple data sources with entropy-based estimation, we construct consistent MRIO tables aligned with India’s official national supply–use tables. The dataset reveals the evolution of domestic supply chains during India’s critical growth period between the global financial crisis and the COVID-19 pandemic. It provides a foundational resource for studies on industrialisation, regional inequality, environmental impacts, and spillover effects across regions.

## Introduction

As the second largest developing country, India has undergone profound structural transformation marked by rapid industrialisation and shifts in production and trade patterns. These developments have made India one of the fastest-growing major economies and an increasingly important player in the global system^1^. Since 2022, it overtook the UK to become the fifth largest economy in the world, following USA, China, Japan and Germany. However, due to its vast geography, India has huge regional economic disparities and highly heterogeneous economic structure across the states^2^. Previous studies have shown that fast economic growth since 1980s aggravated regional disparities, instead of converging^3,4^. The diverging development led to the typical disparity where central states are much poorer. For example, BIMARU states, namely Bihar, Madhya Pradesh, Rajasthan, Odisha and Uttar Pradesh in central, refers to poor economic conditions^1^. In contrast, their neighbour states, such as Haryana, Himachal Pradesh, Delhi, Chandigarh, and Uttarakhand are much richer. Regional economic growth cannot be made without supply chains. Therefore, understanding the transformation of cross-state supply chains is critical for policymakers formulating effective development and equity strategies.

The multi-regional Input-output (MRIO) model is widely used for illustrating regional economic system with highlighting cross-region supply chains^5^. Most of efforts have focused on international level and yielded several international datasets, such as GTAP^6^, WIOD^7,8^, Emerging^9^, and EXIOBASE^10^. Subnational level MRIO is emerging with a growing importance. It has been widely used in environmental and social economic impact assessment^11–14^. However, there are very scarcity at subnational level.

It is particularly in developing countries such as India^15^. To the best of our knowledge, there is only one Indian MRIO model developed by our previous work^1^. The model is developed by non-survey approach, using Flegg Location Quotient (LQ). The model covers 32 states and 50 economic sectors, but only for 2015, which limits the ability to analyse supply chain dynamics during India’s economic transition over the past years. The absence of an updated dataset that accurately represents the evolving supply chain significantly impairs our comprehension of the diverse impacts of India’s economic growth at the regional level in recent years of rapid industrialisation. Meanwhile, one of the key assumptions made in our previous work is that state-level foreign and domestic trade are fully stimulated by optimisation model, without the real-world data, which could lead to the uncertainty and accuracy concern.

To address this gap, we compiled inter-state MRIO tables from 2011 to 2019 using a hybrid approach of both real-world data and entropy-based stimulation. The method has been widely used in subnational level MRIO model development for China^5^ and Russia^16^. The model covers 32 Indian states with 66 sectors over the period, ensuring consistency and compatibility with India’s official national supply-use tables. The dataset integrates official statistics, including annual survey of Industry (ASI) datasets^17^, India’s official national supply-use tables^18^, India inter-state transport trade dataset^19^, Indian state level socioeconomic dataset^20^, and India’s state-level customs data^21^. The period from 2011 to 2019 reflects India fastest economic growth and transition between the recovering period of 2008 financial crisis and economic shock by pandemic in 2020. This dataset could offer a solid basis for diverse economic, social and environmental analyses, especially regarding interregional trade, regional disparity, and the environmental impacts of economic activities.

## Methods

**Fig. 1:**
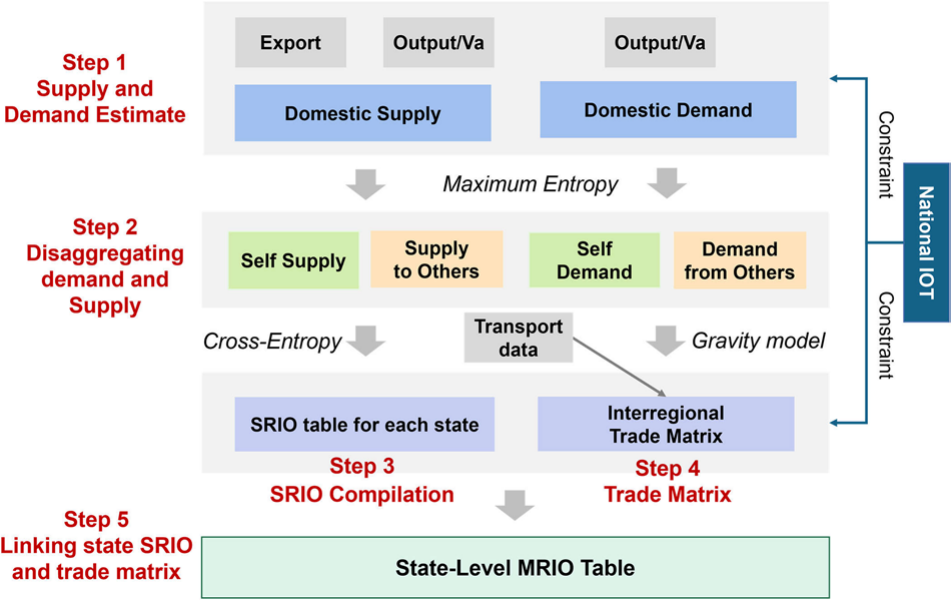
Flowchart of MRIO table construction.

Figure 1 shows the diagram of India state MRIO model construction. India state MRIO tables from 2011 to 2019 adopted a hybrid approach, which combines the official survey data and modelled outcomes^22–24^. The approach is considered better than non-survey approaches, which are widely used due to data limitation^25^. This hybrid approach is to link single region Input-output tables (SRIO) for each state with the inter-state trade flow for each sector, which has been widely used in MRIO construction at different levels^9,10^. The official data include sector-wise economic data for each state from Indiastat and annual survey of Industry (ASI) datasets; State-level export data is sourced from India’s state-level customs datasets. All the data would be benchmarked with the Indian official national input-output table to make sure the consistency. It is worth noting that India publishes Supply–Use Tables (SUTs), which we transform into official symmetric national Input–Output Tables (IOTs) using the Eurostat transformation framework, specifically Model D. Model D is based on the Industry Technology Assumption (ITA), which assumes that all products produced by a given industry are generated using the same production structure. This approach is widely adopted by statistical offices because it remains closely aligned with the observed production data collected from firms^9,26^. This model can ensure industry by industry symmetric table without negative value. Specifically, the calculation is following:

1 $${\bf{D}}={{\bf{V}}\left({\rm{diag}}\left({\rm{q}}\right)\right)}^{-1}$$

2 $${\bf{B}}={{\bf{U}}\left({\rm{diag}}\left({\rm{g}}\right)\right)}^{-1}$$

3 $${\bf{A}}={\bf{B}}\ast {\bf{D}}$$

Where ${\bf{D}}$ indicates market share of each industry in the production of each product, and ${\bf{B}}$ indicates market share of each industry in the production of each product. **V** is the supply matrix, and **U** is the supply matrix. q is the vector of total product output while g is the vector of total industry output. The symmetric A matrix of the IO table can be derived (Eq. 3). Once the coefficients (A) are determined, they are multiplied by the total industry output (g) to create the Z matrix of the IO table.

These data are used to construct single region Input-output tables for each state over the period. In contrast, inter-state trade flow matrix is normally unavailable from national statistical agency, which is stimulated by gravity model^27,28^. Table 1 shows all variables used in the compilation as well as the data sources. The constructed MRIO table comprises 66 sectors and 32 states. However, the original datasets reported in Table 1 differ in both sectoral and regional resolution. At the sectoral level, state-wise value-added data from Indiastat are available only at a 21-sector aggregation. To ensure consistency with the 66-sector MRIO framework, we apply a proportional disaggregation approach. Specifically, sectoral shares derived from the 66-sector Annual Survey of Industries (ASI) are used as weights to split each aggregated 21-sector category into the corresponding detailed sectors. This assumes that the intra-sectoral structure reflected in the ASI data reasonably approximates the detailed composition of state-level aggregates. At the regional level, India officially consists of 36 administrative units (28 states and 8 Union Territories). However, several Union Territories are economically small (e.g., Puducherry and Lakshadweep), which may lead to instability in regional accounts. To enhance statistical robustness and maintain consistency in interregional modelling, selected smaller territories are merged with neighbouring states, resulting in a consolidated 32-region framework. The complete merging scheme and the list of the 32 states are provided in Supplementary Information 1.

**Table 1:** The list of data used in the table construction.

Data	Source	Link
Sector-wise output and value-added for states	Annual survey of industry; Ministry of Statistics & Programme Implementation (MOSPI)^17^	https://microdata.gov.in/NADA/index.php/catalog/ASI/?page=1&sort_order=desc&ps=15&repo=ASI
Gross value added for states	Indiastat^20^	https://www.indiastat.com/
Sectoral export for states	Directorate General of Commercial Intelligence and Statistics (DGCI&S)^21^	https://www.commerce.gov.in/trade-statistics/
Interstate trade data	Directorate General of Commercial Intelligence and Statistics (DGCI&S)^19^	https://www.dgciskol.gov.in/pub_inland.aspx
National supply-use table	Ministry of Statistics & Programme Implementation (MOSPI)^18^	https://www.mospi.gov.in/publications-reports/innerpage/847

Note: Indian Customs data are not publicly available but can be accessed by subscription.

Specifically, Indian MRIO compilation can be divided into following five steps:

### Estimation of domestic demand and supply

The compilation framework is based on the supply and demand balance for an economy. From the supply and demand perspective, the sectoral supply (${s}_{r}^{i}$) for each state can be estimated by Eq. 1:

4 $${s}_{i}^{r}={x}_{i}^{r}-{{ex}}_{i}^{r}$$

Where$\,{x}_{r}^{i}$ refers to the output of sector *i* in state *r*; ${{ex}}_{r}^{i}$ refers to the export of sector *i* in state *r*.$\,{s}_{r}^{i}$ represents the domestic supply of sector *i* for state *r*, indicating the remain output of sector *i* is used domestically. However, the sectorial demand of each state cannot be derived from existing dataset. We estimate them by following equations:

5 $${{td}}_{i}^{{\rm{nation}}}=\sum _{j}{z}_{{ij}}^{{\rm{nation}}}+{f}_{i}^{{\rm{nation}}}$$

Where ${{td}}_{i}^{{\rm{nation}}}$ is the total demand for sector *i* from India national SRIO table, including intermediate demand (${z}_{{ij}}^{{\rm{nation}}}$) and final demand (${f}_{i}^{{\rm{nation}}}$). For domestic demands for any state *n*:

6 $${{td}}_{i}^{n}=\left(\frac{\sum _{j}\left({a}_{{ij}}^{{\rm{nation}}}\times {x}_{j}^{n}\right)}{\left(\frac{\sum _{j}{z}_{{ij}}^{{\rm{nation}}}}{{{td}}_{i}^{{\rm{nation}}}}\right)}\right)$$

7 $${d}_{i}^{n}=\frac{{{td}}_{i}^{n}}{\sum _{n}{{td}}_{i}^{n}}\times {{nd}}_{i}^{{\rm{nation}}}$$

Where ${{td}}_{i}^{n}$ is the preliminary total demand for sector i for state n. $\sum _{j}\left({a}_{{ij}}^{{\rm{nation}}}\times {x}_{j}^{n}\right)$ represents the sum of preliminary intermediate demand for sector i. ${a}_{{ij}}^{{\rm{nation}}}$ is the national technical coefficient for sector i and j. The assumption gives a preliminary estimate of total demand of each state, as the technical coefficient of each state is unknown. This assumption provides a preliminary estimate of total state demand, with the national coefficient matrix serving as a pragmatic prior for regional production structures. The denominator $\left(\frac{\sum _{j}{z}_{i}^{{\rm{nation}}}}{{{td}}_{i}^{{\rm{nation}}}}\right)$ is the ratio of intermediate demand to total demand at national level, initially assuming identical technical coefficient between India state and nation. The total demand for state n for sector i (${d}_{i}^{n}$) is estimated by using preliminary total demand for sector i for state n to distribute ${{nd}}_{i}^{{\rm{nation}}}$ which is the India domestic demand for sector i. The estimated demand for each state is then treated as a distributor to adjust ${{nd}}_{i}^{{\rm{nation}}}$, which is equal to difference between output and foreign export for sector i. This can ensure the consistency between the aggregation of estimated total demand for each state n and national total demand, shown in Eq. 7.

To calculate the supply and demand, output and value-added can be collected from annual survey of Industry (ASI) datasets and the Ministry of Statistics and Programme Implementation (MoSPI), where the former dataset provides the economic metrics for industrial sectors and the later offers the metrics for primary sectors and tertiary sectors. Given the discrepancy between regional survey data and national statistics, the sector-wise output and value-added for each state are benchmarked by the national Input-output table to ensure the consistency between regional data and national data. Foreign export data by state and sector can be sourced from India customs dataset managed by Indian Directorate General of Commercial Intelligence and Statistics (DGCI&S). Foreign imports data by state are not available, and we estimate the state-wise import for sectors by using total demand of each state to distribute aggregated imports. Specifically,

8 $${{IM}}_{i}^{n}={{IM}}_{i}^{{nation}}\times \frac{{{td}}_{i}^{n}}{\sum _{n}t{d}_{i}^{n}}$$

Where ${{IM}}_{i}^{{nation}}$ is the Indian national import for sector *i* derived from Indian national supply use table; ${d}_{i}^{n}$ is the total demand for sector *i* and state n estimated in Eq. 3.

### Disaggregating demand and supply

**Fig. 2:**
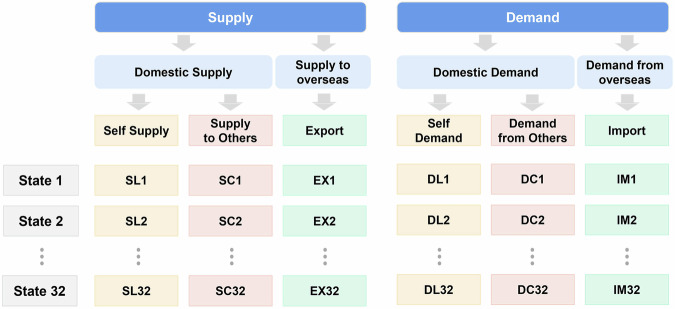
The illustration of a supply-and-demand balancing matrix for all states in India for any sector. Domestic supply for each state consists of self-supply (SL) and supply to other states (SC), whereas domestic demand (D) includes locally supplied demand (DL) and demand from other states (DC).

For a given state, the supply of a product in the state refers to the where the locally produced product used and it can be further categorised into self-supply, supply to other state (domestic export), and foreign export. Similarly, the demand of a state can be defined by where provides the product, which can be further categorised into self-demand, demand from other states (domestic import), and foreign import. Therefore, we can construct an entropy-based optimisation model to estimate disaggregated supply and demand, with following relationships. Because all export and import are known, the equations below only simulate domestic supply and demand. For a certain product, (1) the self-supply is equal to the self-demand for the same state; (2) the sum of self-supply and supply to other states (domestic export) should be equal to the total domestic supply. Correspondingly, (3) the sum of self-demand and demand from other states (domestic import) should be identical with the total domestic demand; (4) the column sum of domestic export for all states should be equal to the column sum of domestic import for all states, as they are traded within same boundary. It is shown in Fig. 2 Mathematically, the maximum entropy (denoted by *E*) to estimate supply and demand is expressed as:

9 $$\max E\left({p}_{n}\right)=-\sum \sum {p}_{n}\cdot \mathrm{ln}{p}_{n}$$

subject to:

$${p}_{n}^{{\rm{SL}}}\times \sum _{n}{s}_{n}={p}_{n}^{{\rm{DL}}}\times \sum _{n}{d}_{n}$$

$$\sum _{n}({p}_{n}^{{\rm{SC}}}\times \sum _{n}{s}_{n})=\sum _{n}{(p}_{n}^{{\rm{DC}}}\times \sum _{n}{d}_{n})$$

$$\sum _{n}{p}_{n}^{{\rm{SL}}}+\sum _{n}{p}_{n}^{{\rm{SC}}}=1$$

$$\sum _{n}{p}_{n}^{{\rm{DL}}}+\sum _{n}{p}_{n}^{{\rm{DC}}}=1$$

$$\left({p}_{n}^{{\rm{SL}}}+{p}_{n}^{{\rm{SC}}}\right)\times \sum _{n}{s}_{n}={s}_{n}$$

$$({p}_{n}^{{\rm{DL}}}+{p}_{n}^{{\rm{DC}}})\times \sum _{n}{d}_{n}={d}_{n}$$

$$\sum _{n}\left(\sum _{n}{s}_{n}\right)\times {p}_{n}^{{\rm{SL}}}+\sum _{n}\left(\sum _{n}{s}_{n}\right)\times {p}_{n}^{{\rm{SC}}}=\sum _{n}{s}_{n}$$

$$\sum _{n}\left(\sum _{n}{d}_{n}\right)\times {p}_{n}^{{\rm{DL}}}+\sum _{n}\left(\sum _{n}{d}_{n}\right)\times {p}_{n}^{{\rm{DC}}}=\sum _{n}{d}_{n}$$

$${p}_{n}^{{\rm{DL}}} > 0$$

In the above, ${p}_{n}$ denotes the probability for state *n*, including the probabilities of domestic supplies (${p}_{n}^{{\rm{SL}}}$,$\,{p}_{n}^{{\rm{SC}}}$) and the probabilities of domestic demands (${p}_{n}^{{\rm{DL}}}$,$\,{p}_{n}^{{\rm{DC}}}$). The last constraint (${p}_{n}^{{\rm{DL}}} > 0$) states that the probability of local demand cannot be zero, which avoids an unrealistic case where all local demands are met by imports from other regions, and all local supplies go to meet the demands of other regions. In other words, this constraint assumes that the local supply prioritises meeting the local demand.

### The state SRIO table compilation

Above steps simulate the domestic supply and demand. With known export and import, we can calculate net export by sector for each state and then construct the SRIO table for each state by employing the cross-entropy model. The SRIO table for state r should meet two conditions. By row, the row sum of intermediate and final demand should be equal to total output minus net export. By column, the column sum of intermediate demand should be equal to total output minus value-added. Mathematically:

10 $$\min \,{\rm{C}}({\rm{P}}\parallel {\rm{Q}})=\sum _{{\rm{i}}}\sum _{{\rm{r}}}{p}_{r}\cdot \mathrm{ln}\left(\frac{{p}_{r}}{e}\right)$$

Subject to:

$$\sum _{{\rm{j}}}{q}_{r}^{{ij}}\,\cdot \,{p}_{r}^{{ij}}={X}_{r}^{j}-{{VA}}_{r}^{j};$$

$$\sum _{{\rm{i}}}{q}_{r}^{{ij}}\,\cdot \,{p}_{r}^{{ij}}=\,{X}_{r}^{i}-{{NE}}_{r}^{i};$$

Where ${p}_{r}^{{ij}}$ represents the unknown distribution dividing known prior distribution, which is the result of the cross-entropy; $e$ is the Natural logarithm. ${X}_{r}^{j}$ represents the total input of sector j for state r, while ${x}_{r}^{i}$ represents the total output of sector *i* for state *r*; ${q}_{r}^{{ij}}$ is the prior distribution containing the matrix of intermediate demand ${z}_{r}^{{ij}}$ and final demand ${f}_{r}^{i}$, showing an economic structure for each state. For the final demand, we first calculate the aggregated final demand by GDP minus ${NE}$, and then multiply the final demand structure derived from national input-output table as the proxy estimate. ${{ne}}_{r}^{i}$ represents the net export of product *i* for province *r*, which is equal to foreign export + domestic export-foreign import-domestic import by product. The prior distribution is derived from India national input-output table, including both intermediate demand and final demand. The rationale behind the assumption is that the prior distribution from the national input-output table shows an average level or benchmark. The state-wise economic structure can be derived from the national economic structure with a modification subject to the state level data. The practice has been widely used in the previous subnational IO table estimation^9,15,16,29^.

### The interstate trade matrix estimation

To obtain the India’s interstate trade flow matrix, we apply the gravity model with the observable trade data between state derived from DGCIS, which improves the accuracy and reliability of the interregional estimates^24,30,31^. The gravity model has been widely adopted in previous MRIO table building^27,29,32–34^. The model assumes that the trade between two regions is the function of supply and demand and the impedance in costs (distance between states). Therefore, the standard gravity model and its logarithmic form is as follows:

11 $${t}_{{rs}}^{i}={G}^{\alpha }\frac{{\left({{e}_{{\rm{ro}}}}^{i}\right)}^{{\beta }_{1}}\times {\left({{m}_{{\rm{os}}}}^{i}\right)}^{{\beta }_{2}}}{{\left({d}_{{rs}}\right)}^{\gamma }}$$

12 $$\mathrm{ln}\,{t}_{{rs}}^{i}=\alpha \ast \mathrm{ln}\,G+{\beta }_{1}\ast \mathrm{ln}\,{{e}_{{\rm{ro}}}}^{i}+{\beta }_{21}\ast \mathrm{ln}\,{{m}_{{\rm{os}}}}^{i}-\gamma \ast \mathrm{ln}\,{d}_{{rs}}$$

Where ${t}_{{rs}}^{i}$ is the trade flow for sector product *i* between state *r* and state *s*; ${{e}_{{\rm{ro}}}}^{i}$ and ${{m}_{{\rm{os}}}}^{i}$ are the supply (or domestic export) from state *r* and the demand (or domestic import) of state *s*, respectively. ${d}_{{rs}}$ is the distance between two states, which is the proxy for transportation costs. *β*_1_ and *β*_2_ represent the weights of the original and destination province. $\gamma$ refers to the friction parameter. In this case, we use the road-transportation inter-state goods from DGCIS as sample data for the shippable commodity. We use the sample data as the trade flow (${t}_{{rs}}^{i}$) to estimate the unknown coefficients (*β*1, *β*2,*γ*). The interstate trade data are only for 27 sectors, which means some sectors in the gravity model have to share the same coefficients. We show the mapping relationship in the appendix. To estimate trade patterns, we use the gravity model to build an initial trade matrix for all commodity sectors.

For non-shippable commodities, such as services or electricity, we assume the coefficients (*β*1, *β*2, *γ*) are equal to 1, where supply * demand /distance. It means the weights for both supply and demand are same. Mathematically:

13 $${t}_{{rs}}^{k}=\frac{{e}_{{\rm{ro}}}\times {m}_{{\rm{os}}}}{{(d}_{{rs}})}$$

The equation is to estimate the initial trade pattern ${t}_{{rs}}^{k}$, where k refers to non-shippable commodities. To reconcile these differences, we apply the RAS procedure to adjust the trade matrices, ensuring consistency with observed domestic export (row) and import (column) totals. The practice has been adopted in previous works^5,15^. However, it is noted that the distances we used in the trade flow estimation are great-circle distances between state centroids. Travel times or generalized transport costs could be a better choice, but it is generally not available.

### linking state SRIO and trade matrix

The final step is to link the state SRIO table generated in Step 3 and trade matrix generated in Step 4. However, state SRIO table was made in type-A input-output table^35^, which means the intermediate and final demands in the state SRIO table include domestic and foreign imports. However, the MRIO table compilation distinguished domestic and imported goods, where we modify the table by purchase coefficient (${{pc}}_{r}^{i}$). Mathematically:

14 $${{pc}}_{r}^{i}=\frac{\left({x}_{r}^{i}-{{ex}}_{r}^{i}-{{so}}_{r}^{i}\right)}{\left({x}_{r}^{i}-{{ex}}_{r}^{i}-{{so}}_{r}^{i}+{{im}}_{r}^{i}+{{do}}_{r}^{i}\right)}$$

15 $${z}_{{rr}}^{{ij}}={{pc}}_{r}^{i}\times {z}_{r}^{{ij}}$$

16 $${f}_{{rr}}^{i}={{pc}}_{r}^{i}\times {f}_{r}^{i}$$

Where ${{pc}}_{r}^{i}$ denotes share of intermediate and final demand met by local product. Applying the identical share to both intermediate and final demand assumes the identical share of imports in the intermediate and final demands in the SRIO table. ${x}_{r}^{i}$ refers to the output of sector *i* in state *r*; ${{ex}}_{r}^{i}$ indicates the foreign export of sector *i* in state *r*; ${{so}}_{r}^{i}$ refers to product *i* supply from state *r* to others; ${{im}}_{r}^{i}$ represents sector *i* imported from foreign countries to state *r*;$\,{{do}}_{r}^{i}$ represents sector *i* required in state *r*.$\,{z}_{{rr}}^{{ij}}$ and ${f}_{{rr}}^{i}$ are intermediate and final demand met by domestic sector *i* for state *r*, respectively. This can generate the diagonal matrix of a MRIO table.

Accordingly, we calculate the proportion of total domestically imported products supplied from each province, defined as regional purchase proportion (RP).

17 $${{rp}}_{{rs}}^{i}=\frac{{t}_{{rs}}^{i}}{\sum _{s}{t}_{{rs}}^{i}}$$

18 $${z\,}_{{rs}}^{{ij}}={{rp}}_{{rs}}^{i}\times {{zn}}_{s}^{{ij}}r\ne s$$

19 $${f}_{{rs}}^{i}{\,=\,{rp}}_{{rs}}^{i}\times {{fn}}_{s}^{i}r\ne s$$

Where ${{rp}}_{{rs}}^{i}$ represents the ratio of domestic imports from state *r* to state *s* for sector *i*; ${t}_{{\rm{jr}}}^{{\rm{i}}}$ refers to the trade from state *j* to state *r* for sector *i*. ${{zn}}_{s}^{{ij}}$ and ${{fn}}_{s}^{i}$ refer to the aggregate domestic imports derived from state SRIO table. ${z}_{{rs}}^{{ij}}$ and final demand ${f}_{{rs}}^{i}$ are intermediate demand and final demand met by domestic imports from state r to state s. This can generate the off-diagonal matrix of a MRIO table.

## Data Records

**Fig. 3:**
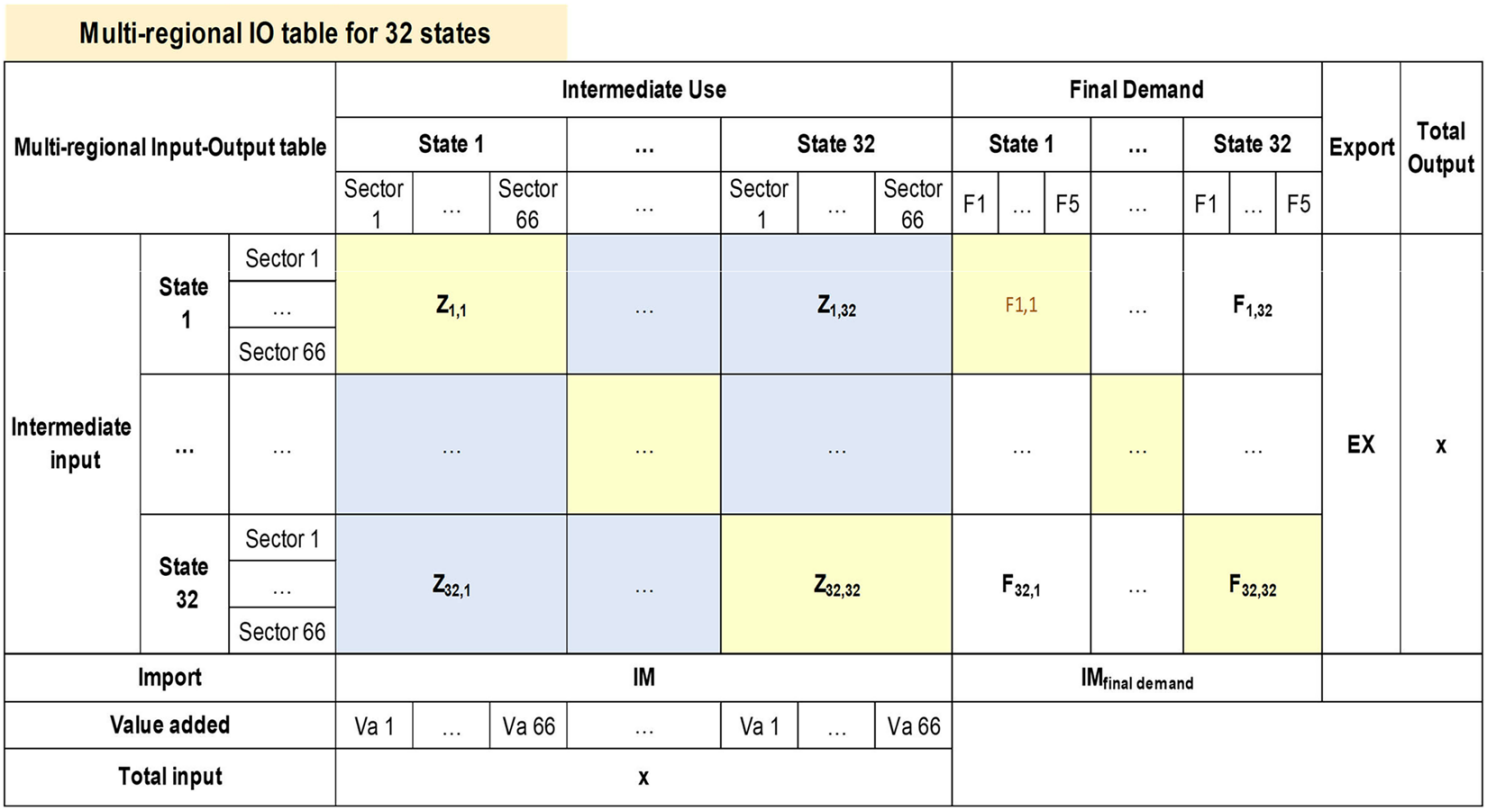
The layout of the India state-level MRIO table.

State MRIO tables illustrate the regional economic structure and interregional supply chains for 32 states with 66 sectors and cover the period from 2011 to 2019. The price unit is Rs Lakh (100 thousand Indian Rupee) at current price. Figure 3 shows the layout of the MRIO tables. For each year, the MRIO table contains an intermediate matrix (2112*2112) for the 66 sectors in 32 states. The final demand of each state consists of 5 categories, including Private Final Consumption Expenditure, Government Final Consumption Expenditure, Gross Fixed Capital Formation, Valuables, and Change in Stocks. The final demand matrix contains 2112*160 vectors for each year. In addition, foreign export contains 2112*1 vectors measuring the export for all 66 sectors in 32 states, while import contains 1*2112 vectors indicating the imports from other countries used by all 66 sectors in 32 provinces. Due to the data limitation, value-added includes one row, with 1*2112 vectors. The MRIO tables can be downloaded from the Carbon Emission Accounts and Datasets for emerging economies (CEADs, www.ceads.net) and figshare^36^. The MRIO table constructed in the paper is only at the state-level, and it can be nested into global MRIO tables for the global scale analysis^37–39^. Due to the optimisation algorithm, the table generated by this method may contain a small discrepancy, defined as the gap between total output and the sum of intermediate demand, final demand, and exports, which is controlled within 5% as the tolerance benchmark.

## Technical Validation

### Compare with 2015 India MRIO dataset

We compare our new India MRIO dataset with 32 states and 66 sectors (Emerging_India) with our previous work^1^ with 33 states and 50 sectors in 2015. To keep the consistency, we adjust two datasets into the format of 32 states and 50 sectors. Specifically, we merge the 66 sectors into 50 sectors for the Emerging_India and merge the 33 states into 32 states for Huang’s India. Following previous work in MRIO table comparison^1^, three indicators measuring the similarity between matrix are employed in the comparison. Namely, the mean absolute deviation (MAD), which measures the absolute distance between each element in the two matrices; the Isard-Romanoff similarity index (DSIM) which uses the relative distance instead of the absolute distance in MAD; the absolute entropy distance (AED), which is based on information theory and refers to the entropy loss between two matrices. It calculates the absolute entropy differences between two matrices. More similar to two matrices, AED is closer to zero. Here, we compare the intermediate demand matrix, representing how the sector’s production requires the other sector’s production. Mathematically:

20 $${MAD}=\frac{1}{m\times n}\sum _{m}\sum _{n}\left|{z}_{{ij}}^{{Emerging}{\rm{\_}}{India}}-{z}_{{ij}}^{{Huan}{g}^{{\prime} }s\,{India}}\right|$$

21 $${DSIM}=\frac{1}{m\times n}\sum _{m}\sum _{n}\frac{\left|{z}_{{ij}}^{{Emerging}{\rm{\_}}{India}}-{z}_{{ij}}^{{Huan}{g}^{{\prime} }s\,{India}}\right|}{\left|{z}_{{ij}}^{{Emerging}{\rm{\_}}{India}}\right|+\left|{z}_{{ij}}^{{Huan}{g}^{{\prime} }s\,{India}}\right|}$$

22 $${AED}=\left|\sum _{i}\sum _{j}{p}_{{ij}}\mathrm{ln}\,{p}_{{ij}}-\sum _{i}\sum _{j}{q}_{{ij}}\,\mathrm{ln}\,{q}_{{ij}}\right|$$

where

23 $${p}_{{ij}}=\frac{{z}_{{ij}}^{{Emergin}{g}_{{India}}}}{\sum _{i}\sum _{i}{z}_{{ij}}^{{Emergin}{g}_{{India}}}}$$

24 $${q}_{{ij}}=\frac{{z}_{{ij}}^{{Huan}g{\prime} s\,{India}}}{\sum _{i}\sum _{i}{z}_{{ij}}^{{Huan}g{\prime} s\,{India}}}$$

Here, ${z}_{{ij}}^{{Emerging\_India}}$ refers to the intermediate demand for sector *j* from sector *i* of Emerging_India; ${z}_{{ij}}^{{Huan}{g}^{{\prime} }{s\; India}}$ denotes the intermediate demand for sector *j* from sector *i* of our previous work.

Table 2 showed the discrepancies of three indicators between Emerging_India and Huang’s work. In terms of MAD, our MRIO table shows that 10 states are similar to them in Huang’s work. MAD gives more weight to the large number, which means it might be more sensitive for the rich or large states which have larger transactions. Our results accordingly show that indicators for small states such as Goa, Sikkim and Tripura are more similar to Huang’s work. However, DSIM measuring the relative distance indicates higher similarity with Huang’s work where 29 states are closer to our previous work. AED compares the matrix in terms of information (or entropy) loss. It indicates a little similarity with our previous work, with an average entropy loss of 46%. There are 6 states showing a small discrepancy. Overall, three indicators might explain a significant difference with Huang’s work, with several states showing larger similarity. However, such comparison is expected to have higher discrepancy, as the data sources and methodology are very different between two datasets.

**Table 2:** Comparison of MAD, DSIM, AED between India dataset in the paper and Huang’s work.

State	MAD	DSIM	AED
Andhra Pradesh	26623	21	48%
Assam	9569	21	73%
Bihar	16945	22	49%
Chhattisgarh	11436	21	30%
Goa	2820	23	22%
Gujarat	76660	21	57%
Haryana	33855	22	35%
Himachal Pradesh	5868	23	81%
Jammu & Kashmir	4702	22	62%
Jharkhand	11123	21	54%
Karnataka	50872	22	63%
Kerala	25033	23	65%
Madhya Pradesh	22716	20	25%
Maharashtra	109086	22	80%
Manipur	666	16	79%
Meghalaya	948	16	44%
Mizoram	528	29	6%
Nagaland	707	18	162%
Orissa	16090	21	60%
Punjab	20546	22	43%
Rajasthan	29835	22	20%
Sikkim	680	17	113%
Tamil Nadu	60731	22	66%
Telangana	24613	22	28%
Tripura	1065	20	4%
Uttar Pradesh	52759	22	59%
Uttrakhand	11334	23	6%
West Bengal	37536	21	57%
A & N Island	256	17	86%
Chandigarh	1560	20	21%
Delhi	28691	24	9%
Puducherry	1530	22	26%

### MAD of India MRIO tables over the period 2011 to 2019

We applied the Mean Absolute Deviation (MAD) to each element of the MRIO tables for the years 2011–2019 (Table 3). The MAD was chained, meaning values in year *t* were compared with those in year *t–1*. The results indicate that the average absolute distance between years remained stable and steadily increasing, suggesting a consistent structural pattern across the nine-year period. A noticeable increase in structural deviation is observed between 2016 and 2017, which is likely associated with the economic slowdown following the demonetisation policy implemented in 2016. This impact appears to be less pronounced in the Northeast states, potentially due to their relatively underdeveloped economic base.

**Table 3:** Chained MAD results across 2011 to 2019 for 32 states in India.

States	2011–2012	2012–2013	2013–2014	2014–2015	2015–2016	2016–2017	2017–2018	2018–2019	2018–2019
Andhra Pradesh	5.99	7.26	7.45	7.28	11.02	11.44	8.69	6.94	5.99
Assam	6.40	8.45	7.35	6.97	10.86	11.44	8.45	7.11	6.40
Bihar	6.17	7.57	6.60	6.71	10.11	10.52	8.39	6.71	6.17
Chhattisgarh	6.00	7.78	7.51	6.42	10.62	10.47	8.10	6.96	6.00
Goa	7.24	9.32	9.67	9.13	11.97	11.89	9.18	7.75	7.24
Gujarat	5.60	8.14	7.76	6.52	12.66	11.70	8.35	6.25	5.60
Haryana	5.05	7.39	8.09	7.72	11.57	11.43	8.41	5.45	5.05
Himachal Pradesh	6.08	7.63	8.13	7.68	11.42	10.99	8.29	6.63	6.08
Jammu & Kashmir	6.17	7.98	6.94	6.48	9.63	10.04	7.48	6.22	6.17
Jharkhand	6.42	6.93	6.60	6.09	10.04	10.67	8.32	6.73	6.42
Karnataka	5.36	7.27	7.57	6.52	10.71	11.04	8.31	6.32	5.36
Kerala	5.37	7.21	7.16	6.52	10.84	11.56	8.98	7.18	5.37
Madhya Pradesh	6.09	7.53	7.78	6.68	10.39	10.39	7.75	7.09	6.09
Maharashtra	4.73	6.83	7.44	6.56	11.05	11.25	8.83	6.85	4.73
Manipur	5.30	5.99	6.80	5.69	8.75	8.85	7.42	6.19	5.30
Meghalaya	4.31	6.35	6.59	5.58	8.58	9.08	7.00	6.54	4.31
Mizoram	3.46	5.43	6.82	5.05	7.29	8.24	6.37	5.84	3.46
Nagaland	3.84	5.66	7.03	4.86	8.35	8.32	6.45	6.77	3.84
Orissa	6.18	7.11	6.95	6.38	10.25	11.57	7.95	6.69	6.18
Punjab	5.06	7.17	7.39	7.05	11.23	11.45	7.94	6.39	5.06
Rajasthan	5.37	7.89	8.09	7.19	11.08	11.19	8.00	6.35	5.37
Sikkim	4.94	6.63	9.19	8.38	11.43	11.09	9.40	9.06	4.94
Tamil Nadu	5.16	7.55	8.12	7.06	11.42	11.56	8.70	7.11	5.16
Telangana	4.63	7.92	7.91	7.20	11.32	11.80	8.71	7.10	4.63
Tripura	5.45	7.83	8.09	7.42	10.35	11.41	8.35	7.86	5.45
Uttar Pradesh	5.05	7.20	8.17	7.07	11.30	11.44	8.81	7.12	5.05
Uttrakhand	5.06	7.49	8.33	7.27	11.16	11.69	8.63	6.91	5.06
West Bengal	5.81	7.85	7.48	6.86	11.50	11.50	8.37	6.56	5.81
A & N Island	4.05	5.41	8.67	7.30	10.13	10.83	9.39	7.55	4.05
Chandigarh	7.70	9.85	9.19	7.46	11.90	12.20	9.78	8.37	7.70
Delhi	6.61	8.65	9.64	9.44	13.77	13.49	11.44	8.84	6.61
Puducherry	8.24	9.33	10.66	8.63	12.60	12.31	10.53	9.37	8.24

### Technical Coefficients and Trade stability

**Fig. 4:**
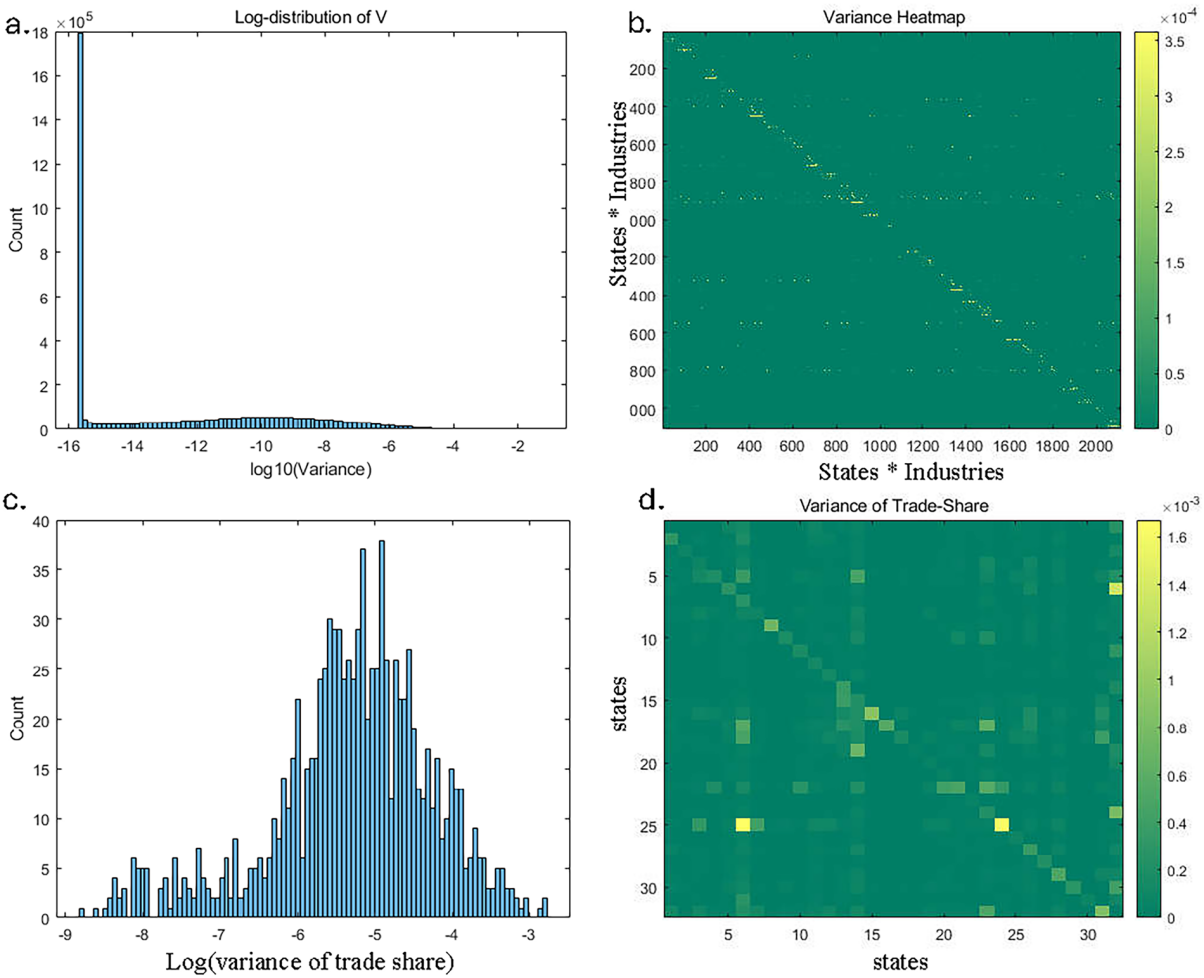
(**a**) Histogram of the element-wise variance (log scale). (**b**) Heatmap of the variance. (**c**) Distribution of the logarithmic variance of trade share. (**d**) Variance of trade share across 32 Indian states.

We compute the element-wise variance of the technical coefficients over a 9-year period (2011–2019). Specifically, variance is calculated for each individual coefficient using nine annual observations per matrix cell. In total, the matrix contains more than four million elements. The mean variance is 2.58 × 10^−6^, and the distribution of variance in logarithmic form ranges mainly between –16 and –5 (Fig. 4a). Figure 4b shows that most elements with relatively high variance appear along the diagonal, suggesting that core industrial technologies have undergone notable development and transformation over the ten-year period.

For trade share, we compute annual trade shares for all 32 states from 2011 to 2019 to examine their stability. Trade share is defined as the ratio of total outflow to total output for each state. The results indicate overall stability, with gradual changes consistent with economic growth and structural development. The logarithmic variance of trade share is relatively low, ranging from –2.78 (≈0.001) to –8.79 (≈1.62 × 10^−9^), and is predominantly concentrated around −5.1 (≈7.94 × 10^−6^) (Fig. 4c). Notably, trade from Telangana (no. 25) to Goa (no. 6) and to Tamil Nadu (no. 24) shows slightly higher variance (Fig. 4d). Over time, trade to Goa declined, whereas trade to Tamil Nadu increased.

### Sensitivity of Initial Trade Matrix of non-shippable Services

In estimating trade flows for non-shippable sectors, our baseline model constructs an initial trade matrix by applying equal weights to supply and demand constraints, with geographical distance incorporated as a penalty factor. This distance-weighted prior matrix is subsequently balanced to match observed margins using the RAS technique, while flows for shippable sectors are derived directly from the gravity model specified in Eq. 7. To validate the robustness of the distance-dependent assumption for non-shippable sectors, we conduct a sensitivity analysis by replacing the distance-weighted prior with an even prior that is, an initial matrix assuming a uniform distribution of trade. A comparison based on the MAD reveals that estimates for tertiary (non-shippable) sectors are significantly less sensitive to this change in prior than those for shippable goods. This is reflected in the aggregate MAD values: 442 for non-shippable sectors versus 2035 for shippable sectors. Within non-shippable sectors, Construction is a notable outlier, with a MAD of 3626, indicating that its estimated flows remain highly sensitive to distance. Excluding Construction, the average MAD for the remaining non-shippable sectors drops to 314. The lower MAD for non-shippable sectors (excluding Construction) suggests that their estimated trade patterns are less sensitive to the choice of prior distribution and, in effect, closer to an even distribution than those of shippable goods.

### Compare with India national input-output table

**Fig. 5:**
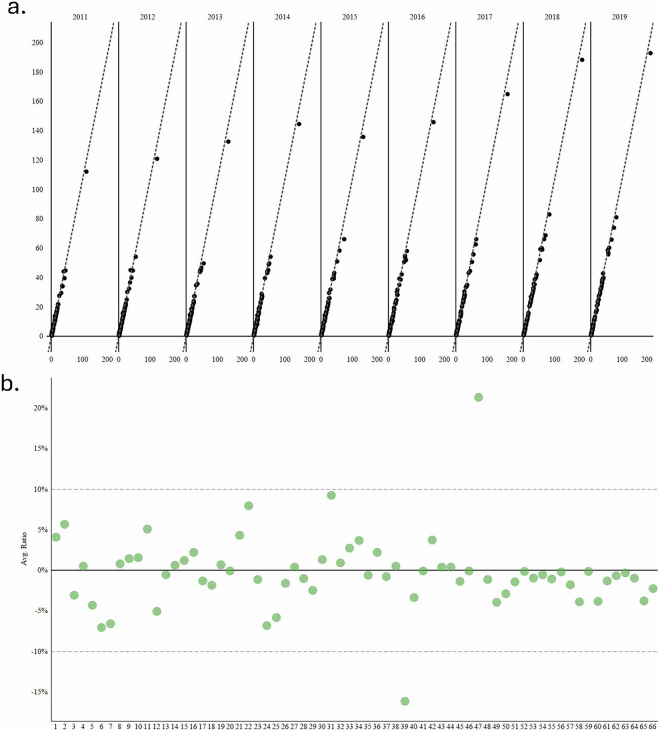
(**a**) Comparison between national SRIO tables and MRIO tables in terms of intermediate input. The dashed line is the 45-degree line (unit: 1 million Rk); (**b**) The average gap between two datasets by sector in terms of difference rate.

We further compare our MRIO table over 2011 to 2019 with their national SRIO tables (Fig. 5a). However, India statistical agency provides the supply-use table (SUT), which have been converted into Type A input-output table, where imports are included in the intermediate and final demands. To compare with MRIO table which is Type B table (imports are separated from intermediate and final demands), the assumption of identical shares of imports in intermediate and final demand by sector has been applied to India IO table and convert it to non-competitive IO table. This assumption has been also applied to MRIO table compilation (Eq. 8). During our compilation process, the key elements of a MRIO table such as output and value-add have been calibrated and benchmarked with the corresponding variables in India national IOT. But sectoral imports by states are estimated based on the demands which is calculated in Eqs. 3, 4, due to no import data. However, the treatment of import including its estimates and the conversion could lead to uncertainty. Therefore, we compare the domestic intermediate input (output minus value-added and imports) between MRIO and SRIO tables from 2011 to 2019. The results show that the gap between MRIO and SRIO tables in terms of domestic intermediate input is roughly small, with averagely 0.3% differences between two datasets. Most of sectors are ± 10% in all nine years, except for two sectors (Fig. 4b). Other Manufacturing (S39) and Water Transport (S47) are outliners, with the average discrepancy rate of −16% and 21% respectively. Other Manufacturing refers to gems or jewellery and water transport refers to the fee for shipping. These two sectors are of highly imported, with 70% of the supply imported overseas. Given the assumptions in import estimates, it is understandable that the sectors with higher imports would be more sensitive. The discrepancy can be minimised if more detailed imports data are available to improve the performance.

## Limitation and Discussion

Constructing subnational MRIO tables is a labor-intensive and time-consuming task that faces significant challenges: (1) the absence of regional input–output tables (SRIOs), and (2) the lack of interregional trade matrices. These constraints compel researchers to rely on non-survey or partial-survey estimation methods, among which the most widely used are location quotient (LQ) methods^40,41^, the commodity balance (CB) method^42^, and the cross-hauling adjusted regionalisation method (CHARM)^43,44^. However, each of these existing approaches has notable limitations.

The location quotient (LQ) method is widely used because it is computationally simple. It is based on the core assumption that regional technical coefficients can be derived by adjusting national technical coefficients. Under this assumption, the method can be used to estimate regional intermediate use and final demand. However, the LQ method is fundamentally grounded in the Type-B input–output framework, which assumes that both intermediate and final demand exclude imports. In contrast, India’s national SRIO tables are Type-A tables. Therefore, the Type-B framework must be converted into a Type-A framework before the LQ method can be applied^35^. The approach typically assumes that imported and domestically produced goods share the same input structure, but this overlooks differences in sectoral import dependence and imbalances in import structure. The downscaling of final demand relies on strong assumptions, such as using value added as the distribution basis, even though value added does not accurately reflect local consumption patterns^24^. For these reasons, the LQ method is not suitable for direct application to the Indian context. In contrast, the commodity balance (CB) method is more suitable for Type-A tables. However, this approach prevents the CB method from distinguishing between imports and exports, making it impossible to construct a complete SRIO table. To address this limitation, Kronenberg improved the CB framework and proposed the cross-hauling adjusted regionalisation method^45^. This method allows for the distinction between exports and imports. However, it still cannot distinguish international trade from domestic trade. Therefore, it remains insufficient for constructing subnational MRIO tables that require detailed interregional trade information. Subsequently, Többen and Kronenberg extended CHARM to estimate domestic trade flows. But, the modified model may underestimate cross-hauling^44^. The method assumes that the cross-hauling shares of states are identical to the national averages, whereas the economic structures of these states are highly heterogeneous.

In this context, our approach of the entropy-based model offers significant advantages. It can generate the least biased estimates under given constraints, even when data is incomplete and information is limited. Jaynes proposed the principle of maximum entropy: among all probability distributions that satisfy the given constraints, the distribution with the highest entropy should be chosen^46^. This principle implies maintaining maximum uncertainty regarding unknown information while fully utilizing the information that is known. In other words, a maximum entropy distribution provides the least-biased estimate that is most consistent with the available but incomplete information. In this study, we extend the CB method by incorporating a maximum entropy model to estimate interregional trade^15^, aiming to construct a MRIO table that balances accuracy and completeness. Compared with existing methods, our approach distinguishes exports from imports, allows reasonable allocation of domestic trade flows, and efficiently utilizes limited data to reduce systematic bias arising from data gaps or strong assumptions.

## Supplementary information

SUPPLEMENTARY

## Data Availability

Indian MRIO Table can be derived from 10.6084/m9.figshare.28736762.

## Code availability

The programs used in the data generation is based on MATLAB and GAMS. The code can be found in https://github.com/HeranZheng/India-MRIO.

## References

[1] Huang, Q. *et al*. Heterogeneity of consumption-based carbon emissions and driving forces in Indian states. *Advances in Applied Energy* **4**, 10.1016/j.adapen.2021.100039 (2021).

[2] Nirola, N., Sahu, S. & Choudhury, A. Fiscal decentralization, regional disparity, and the role of corruption. *The Annals of Regional Science* **68**, 757–787, 10.1007/s00168-021-01102-w (2022).

[3] Kurian, N. J. Widening Regional Disparities in India: Some Indicators. *Economic and Political Weekly* **35**, 538–550 (2000).

[4] Jose, A. India’s regional disparity and its policy responses. *Journal of Public Affairs* **19**, 10.1002/pa.1933 (2019).

[5] Zheng, H. *et al*. Chinese provincial multi-regional input-output database for 2012, 2015, and 2017. *Scientific Data* **8**, 244 (2021). 34552097 10.1038/s41597-021-01023-5 PMC8458474

[6] Peters, G. P., Andrew, R. & Lennox, J. Constructing an Environmentally-Extended Multi-Regional Input–Output Table Using the Gtap Database. *Economic Systems Research* **23**, 131–152, 10.1080/09535314.2011.563234 (2011).

[7] Dietzenbacher, E., Los, B., Stehrer, R., Timmer, M. & de Vries, G. The Construction of World Input–Output Tables in the Wiod Project. *Economic Systems Research* **25**, 71–98, 10.1080/09535314.2012.761180 (2013).

[8] Timmer, M. P., Dietzenbacher, E., Los, B., Stehrer, R. & de Vries, G. J. An Illustrated User Guide to the World Input–Output Database: the Case of Global Automotive Production. *Review of International Economics* **23**, 575–605, 10.1111/roie.12178 (2015).

[9] Huo, J. *et al*. Full-scale, near real-time multi-regional input-output table for the global emerging economies (EMERGING). *Journal of Industrial Ecology* **26**, 1218–1232 (2022).

[10] Wood, R. *et al*. Global Sustainability Accounting—Developing EXIOBASE for Multi-Regional Footprint Analysis. *Sustainability* **7**, 138–163, 10.3390/su7010138 (2014).

[11] Xia, C. *et al*. Outsourced carbon mitigation efforts of Chinese cities from 2012 to 2017. *Nature Cities* **1**, 480–488 (2024).

[12] Zheng, H. *et al*. Leveraging opportunity of low carbon transition by super-emitter cities in China. *Science Bulletin* **68**, 2456–2466, 10.1016/j.scib.2023.08.016 (2023). 37620230 10.1016/j.scib.2023.08.016

[13] Zheng, H. *et al*. Rising carbon inequality and its driving factors from 2005 to 2015. *Global Environmental Change* **82**, 10.1016/j.gloenvcha.2023.102704 (2023).

[14] Kan, S. *et al*. Risk of intact forest landscape loss goes beyond global agricultural supply chains. *One Earth* **6**, 55–65, 10.1016/j.oneear.2022.12.006 (2023).

[15] Zheng, H. *et al*. Entropy-based Chinese city-level MRIO table framework. *Economic Systems Research* **34**, 519–544 (2022).

[16] Li, J. *et al*. Consumption‐Based Carbon Emissions of 85 Federal Entities in Russia. *Earth’s Future* **12**, 10.1029/2023ef004323 (2024).

[17] Ministry of Statistics and Programme Implementation. *Annual Survey of Industries 2011–2019*, https://microdata.gov.in/NADA/index.php/catalog/ASI/?page=1&sort_order=desc&ps=15&repo=ASI (2022).

[18] Ministry of Statistics and Programme Implementation. *Annual Survey of Industries 2011–2019*, https://www.mospi.gov.in/publications-reports/innerpage/847 (2022).

[19] Directorate General of Commercial Intelligence and Statistics. *DGCI&S trade database*. https://www.dgciskol.gov.in/pub_inland.aspx (2023).

[20] Datanet India Pvt. Ltd. Indiastat database. https://www.indiastat.com (2023).

[21] Directorate General of Commercial Intelligence and Statistics. *DGCI&S trade database*. https://www.commerce.gov.in/trade-statistics/ (2023).

[22] Wang, Y., Geschke, A. & Lenzen, M. Constructing a Time Series of Nested Multiregion Input–Output Tables. *International Regional Science Review* **40**, 476–499, 10.1177/0160017615603596 (2015).

[23] Zheng, H. *et al*. Linking city-level input-output table to urban energy footprint: Construction framework and application. *Journal of Industrial Ecology* **23**, 781–795 (2019).

[24] Jahn, M. Extending the FLQ formula: a location quotient-based interregional input–output framework. *Regional Studies* **51**, 1518–1529 (2016).

[25] Hermannsson, K. Beyond Intermediates: The Role of Consumption and Commuting in the Construction of Local Input–Output Tables. *Spatial Economic Analysis* **11**, 315–339, 10.1080/17421772.2016.1177194 (2016).

[26] Kagawa, S. The Sustainability Practitioner’s Guide to Input–Output Analysis. *Economic Systems Research* **24**, 225–227, 10.1080/09535314.2011.590126 (2012).

[27] Cai, M. A calibrated gravity model of interregional trade. *Spatial Economic Analysis* **18**, 89–107, 10.1080/17421772.2022.2081715 (2022).

[28] Mi, Z. *et al*. A multi-regional input-output table mapping China’s economic outputs and interdependencies in 2012. *Scientific Data* **5**, 10.1038/sdata.2018.155 (2018). 10.1038/sdata.2018.155 PMC6080495 30084849

[29] Zhao, Q. *et al*. An inter-regional input-output table series of China from 1987–2017 with integrated carbon emission data. *Scientific Data* **11**, 10.1038/s41597-024-04263-3 (2024). 10.1038/s41597-024-04263-3 PMC11655632 39695185

[30] Yamada, M. Construction of a multi-regional input-output table for Nagoya metropolitan area, Japan. *Journal of Economic Structures* **4**, 10.1186/s40008-015-0022-7 (2015).

[31] Nakano, S. & Nishimura, K. A nonsurvey multiregional input–output estimation allowing cross-hauling: partitioning two regions into three or more parts. *The Annals of Regional Science* **50**, 935–951, 10.1007/s00168-012-0521-5 (2012).

[32] Liu, W., Li, X., Liu, H., Tang, Z. & Guan, D. Estimating inter-regional trade flows in China: A sector-specific statistical model. *Journal of Geographical Sciences* **25**, 1247–1263, 10.1007/s11442-015-1231-6 (2015).

[33] Pan, C. *et al*. Structural Changes in Provincial Emission Transfers within China. *Environmental Science & Technology* **52**, 12958–12967, 10.1021/acs.est.8b03424 (2018). 30339021 10.1021/acs.est.8b03424

[34] Cai, M. Doubly constrained gravity models for interregional trade estimation. *Papers in Regional Science* **100**, 455–475, 10.1111/pirs.12581 (2021).

[35] Wang, Y. An industrial ecology virtual framework for policy making in China. *Economic Systems Research* **29**, 252–274 (2017).

[36] Zheng, H. *et al*. India Multi- Regional Input-output Dataset for 32 States from 2011 to 2019, 10.6084/m9.figshare.28736762 (2026). 10.1038/s41597-026-07227-x PMC13316070 42031806

[37] Zheng, H. *et al*. Regional determinants of China’s consumption-based emissions in the economic transition. *Environmental Research Letters* **15**, 10.1088/1748-9326/ab794f (2020).

[38] Meng, J. *et al*. The role of intermediate trade in the change of carbon flows within China. *Energy Economics* **76**, 303–312, 10.1016/j.eneco.2018.10.009 (2018).

[39] Feng, K. *et al*. Outsourcing CO2 within China. *Proceedings of the National Academy of Sciences* **110**, 11654–11659, 10.1073/pnas.1219918110 (2013). 10.1073/pnas.1219918110 PMC3710878 23754377

[40] Bonfiglio, A. & Chelli, F. Assessing the behaviour of non-survey methods for constructing regional input–output tables through a Monte Carlo simulation. *Economic Systems Research* **20**, 243–258 (2008).

[41] Kowalewksi, J. Regionalization of national input–output tables: Empirical evidence on the use of the FLQ formula. *Regional Studies* **49**, 240–250 (2015).

[42] Miller, R. E. & Blair, P. D. *Input-output analysis: foundations and extensions*. (Cambridge university press, 2009).

[43] Kronenberg, T. Regional input-output models and the treatment of imports in the European System of Accounts (ESA). *Jahrbuch für Regionalwissenschaft* **32**, 175–191 (2012).

[44] Többen, J. & Kronenberg, T. H. Construction of multi-regional input–output tables using the charm method. *Economic systems research* **27**, 487–507 (2015).

[45] Kronenberg, T. Construction of regional input-output tables using nonsurvey methods: the role of cross-hauling. *International regional science review* **32**, 40–64 (2009).

[46] Jaynes, E. T. Information theory and statistical mechanics. *Physical review* **106**, 620 (1957).

